# Risk factors for rectal lymphogranuloma venereum in gay men: results of a multicentre case-control study in the UK

**DOI:** 10.1136/sextrans-2013-051404

**Published:** 2014-02-03

**Authors:** N Macdonald, A K Sullivan, P French, J A White, G Dean, A Smith, A J Winter, S Alexander, C Ison, H Ward

**Affiliations:** 1 Department of Infectious Disease Epidemiology, School of Public Health, Imperial College London, London, UK; 2 Chelsea and Westminster NHS Foundation Trust, London, UK; 3 Mortimer Market Centre, Central and North West London NHS Foundation Trust, London, UK; 4 Guy's and St Thomas’ NHS Foundation Trust, London, UK; 5 Brighton & Sussex University Hospitals NHS Trust, Brighton, UK; 6 Jefferiss Wing Centre for Sexual Health, Imperial College Healthcare NHS Trust, London, UK; 7 Sandyford Sexual Health Services, Glasgow, UK; 8 Sexually Transmitted Bacterial Reference Unit, Public Health England, London, UK

**Keywords:** Chlamydia Trachomatis, Lymphogranuloma Venereum, Gay Men, Sexual Behaviour, HIV

## Abstract

**Objective:**

To identify risk factors for rectal lymphogranuloma venereum (rLGV) in men who have sex with men (MSM).

**Design:**

A case-control study at 6 UK hospitals compared MSM with rLGV (cases) with rLGV-negative controls: MSM without potential rLGV symptoms (CGa) and separately, MSM with such symptoms (CGs).

**Methods:**

Between 2008 and 2010, there were 90 rLGV cases, 74 CGa and 69 CGs recruited. Lifestyles and sexual behaviours in the previous 3 months were reported using internet-based computer-assisted self-interviews. Logistic regression was used to investigate factors associated with rLGV.

**Results:**

Cases were significantly more likely to be HIV-positive (89%) compared with CGa (46%) and CGs (64%). Independent behavioural risks for rLGV were: unprotected receptive anal intercourse (adjusted OR (AOR)10.7, 95% CI 3.5 to 32.8), fisting another (AOR=6.7, CI 1.8 to 25.3), sex under the influence of gamma-hydroxybutyrate (AOR=3.1, CI 1.3 to 7.4) and anonymous sexual contacts (AOR=2.7, CI 1.2 to 6.3), compared with CGa; unprotected insertive anal intercourse (AOR=4.7, CI 2.0 to 10.9) and rectal douching (AOR=2.9 CI 1.3 to 6.6), compared with CGs. An incubation period from exposure to symptoms of 30 days was indicated.

**Conclusions:**

Unprotected receptive anal intercourse is a key risk factor for rectal LGV with the likelihood that rectal-to-rectal transmission is facilitated where insertive anal sex also occurs. The association between HIV and rLGV appears linked to HIV-positive men seeking unprotected sex with others with the same HIV status, sexual and drug interests. Such men should be targeted for frequent STI screening and interventions to minimise associated risks.

## Introduction

An outbreak of lymphogranuloma venereum (LGV) affecting men who have sex with men (MSM) has been recognised since 2003.^1^ Subsequently, cases have been reported from other countries in Western Europe, North America and Australasia.^2–4^ LGV is a sexually transmitted infection (STI) caused by the L-serovars of *Chlamydia trachomatis* (Ct), and while routine Ct tests can detect LGV, identification of specific LGV serovars requires additional subtyping. As many countries do not have access to such resources our global understanding of this infection is restricted.

Where testing and surveillance have been available, consistent features of LGV infections in MSM have been observed. The majority occur in HIV-positive MSM with reported seropositivity rates of 58–100%,^5^ ^6^ and coinfections with other STIs are frequent.^7^ The majority of cases manifest with an acute rectal syndrome, a minority present with anogenital ulcers or buboes of the inguinal/urethral syndrome.^8^ ^9^ However, LGV subtyping is usually only performed on men with symptomatic Ct proctitis,^10^ leading to concerns that inguinogenital and asymptomatic rectal infections may be missed. Symptomatic and asymptomatic pharyngeal LGV infections have also been reported.^11^

Case-finding studies of MSM attending sexual health clinics found rates of LGV rectal infection ranging from 0.2%^12^ to 1.2%,^7^ with the proportion of asymptomatic cases from 5%^13^ to 27%.^7^ Few, if any, urogenital LGV infections were identified in studies that tested Ct-positive urethral samples. Retrospective testing of urethral samples from 341 MSM diagnosed with rectal LGV in Amsterdam between 2008 and 2010 revealed 2% had concurrent urethral LGV and among 59 contacts of the rectal (index) cases, 7% had urethral LGV.^8^

Of the risk factor studies conducted previously, two have examined clinic records^14^ ^15^ but were inconclusive about sexual behaviour. One that included a patient questionnaire found that LGV proctitis in MSM was associated with anal enema use and high-risk sexual behaviour.^16^

Despite a decade of observation many clinical and epidemiological questions remain unanswered: what are the exact modes and risks of transmission given the striking imbalance between rectal and genital infections? To what extent does behaviour or biological susceptibility account for the high levels of HIV coinfection observed? We conducted a case-control study to further explore risk factors for acquisition of LGV.

## Methods

### Study design

A prospective multicentre case-control study was conducted between August 2008 and December 2010 at genitourinary medicine, HIV and specialist (eg, dedicated MSM) clinics of six hospitals in London, Brighton and Glasgow. Centres were selected on the basis of high LGV caseloads. All provide open-access, free-of-charge examination and treatment for STIs.

National ethics committee approval was granted for the study (07/H0712/156) and individual informed patient consent obtained.

### Participant selection

Cases were MSM with confirmed rectal LGV (rLGV). For each case recruited at a participating centre, controls were sought from among eligible patients attending the same clinic in the same week as the case, but were otherwise unmatched. Eligibility for controls included reporting anogenital sex with another man in the previous 3 months and being confirmed rLGV negative. Two types of controls were recruited for each case: symptomatic (CGs), with potential rLGV symptoms (proctitis or anal ulceration), and asymptomatic, without potential rLGV symptoms (CGa). In order to objectify proctitis or ulceration, proctoscopy was carried out on all subjects with rectal symptoms, unless there were clinical contraindications, or if the patient declined. Clinical examinations and STI screening were performed according to local clinic protocols. Ct-positive samples were tested for LGV by the Sexually Transmitted Bacteria Reference Laboratory or the Scottish Bacterial Sexually Transmitted Infections Laboratory, using LGV-specific real-time PCR assays.^17^

## Data collection

### Self-reported patient data

Subjects completed a Computer Assisted Self-Interview (CASI) in a private space in the clinic or were offered a link to the survey by email for home completion. The CASI was designed using Snap Survey software and deployed using the ISO/IEC 27001 certified Snap webhost service.^18^ A study number was assigned by the clinic; no personally identifiable information was requested. Questions probed sociodemographics, HIV and STI testing histories, detailed sexual behaviours, alcohol and substance use in the previous 3 months, prior awareness of LGV and related health promotion, and amenability to health promotion interventions. Details of an event where LGV infection may have been acquired were asked of cases who thought they could identify one. Completion times averaged 20 min.

Of the 261 patients enrolled in the study and tested for rectal Ct/LGV, 233 patient surveys were returned (89%). Loss of internet connection and time constraints at the clinics, and failure to complete the CASI at home by those electing to be emailed the link contributed to the shortfall in patient surveys. The overall response rate for recruitment to the study was 84% (78% for cases, 88% for CGa and 87% for CGs).

### Clinical data

Summary demographics, symptoms at presentation, details of the STI screening tests performed, treatments prescribed, HIV and STI testing histories, and contact tracing outcomes were reported by clinicians through a web-based report form. The data from these surveys were matched to the patient surveys through the study number.

### Statistical analysis

Data were analysed using Stata 12.^19^ Continuous variables were initially explored using t tests. Missing data were rare (<1%) and were omitted. Analyses were conducted separately comparing cases with CGa and CGs. Logistic regression was used to obtain crude and adjusted ORs, 95% CIs and p values. Multivariable analysis considered variables within related strata, retaining those at p<0.05 for wider comparisons. Narrative accounts of potential LGV acquisition events were summarised.

## Results

Questionnaire responses from 90 rLGV cases were compared with those from 74 CGa and 69 CGs. Sixty-seven per cent of cases were recruited from genitourinary medicine clinics, 27% from HIV clinics and 7% from specialist clinics. Similar proportions of the two control groups were recruited from these settings (table 1). Initial presentation because of STI symptoms was reported for 80% of cases and 88% of CGs (p=0.016). Thirteen per cent of cases were identified in the course of a routine check-up, as were 74% of CGa (p<0.001).

**Table 1:** Sociodemographic and clinical data of LGV cases and control groups

Characteristic	Category	Cases (%)	Asymptomatic controls (%)	p Value	Symptomatic controls (%)	p Value
Number		90 (100)	74 (100)		69 (100)	
Clinic-reported						
Recruitment setting	GUM clinic	60 (67)	46 (62)	0.596*	50 (72)	0.412*
	HIV clinic	24 (27)	22 (30)		16 (23)	
	other	6 (7)	6 (8)		3 (4)	
Reason attended	Symptoms	72 (80)	3 (4)	<0.001*	61 (88)	0.491*
	Routine	12 (13)	55 (74)		2 (3)	
	other	6 (7)	16 (22)		6 (9)	
HIV status	HIV+	80 (89)	34 (46)	<0.001	48 (70)	0.003
Self-reported						
Age at interview	Mean±SD	39 ±8	39 ± 10		38 ± 12	
	Median [IQR]	39 [34–44]	38 [33–34]	0.390†	38 [29–45]	0.281†
Ethnicity	White	78 (87)	62 (84)	0.604	55 (80)	0.243
Country of birth	UK	48 (54)	36 (49)	0.550	37 (54)	0.971
First language	English	61 (69)	45 (65)	0.468	48 (70)	0.927
Education	Degree	55 (61)	39 (53)	0.324	40 (58)	0.689
Occupation	Employed or student	76 (85)	65 (88)	0.650	55 (80)	0.348
Sexual identity	Gay	75 (83)	55 (74)	0.159	58 (84)	0.903
Relationship status	Single	43 (48)	40 (54)	0.424	39 (57)	0.275
Years since first:						
Sex with male	Mean±SD	22 ± 8	21 ±10		22 ± 13	
	Median [IQR]	22 [17–29]	20 [14–26]	0.136†	21 [11.5–29]	0.375†
Anal sex with male	Mean±SD	20 ± 8	17 ±10		19 ± 12	
	Median[IQR]	19 [13–26]	16 [10–23]	0.032†	18 [9–26]	0.408†
Attended STI clinic	Mean±SD	14 ± 8	10 ±8		14 ± 12	
	Median [IQR]	14 [9–21]	8.5 [5–13]	<0.001†	11 [4–20]	0.124†
STI history	HIV	80 (89)	34 (46)	<0.001	47 (68)	0.002
	Syphilis	46 (51)	16 (22)	<0.001	32 (46)	0.554
	Hepatitis C	17 (19)	6 (8)	0.069‡	6 (9)	0.110‡
	Herpes	31 (34)	15 (21)	0.046	25 (36)	0.815

*p Value for trend.

†Wilcoxon rank sum test for medians.

‡Fishers exact.

### Characteristics of participants

The median age of cases was 39 years (range 22–56 years). Both control groups were similar to cases in age, the proportion born in the UK, white ethnicity, speaking English as a first language, employment status and educational attainment (table 1).

Eighty-three per cent of cases identified as ‘gay’, with a median of 19 years since first anal intercourse with a man, and 14 years since first attending an HIV/Sexual Health clinic. CGs controls were similar to cases in these respects, as were CGa apart from having significantly fewer years since first anal intercourse (median 16, p=0.032) and since first attending a sexual health clinic (median 8.5, p<0.001).

Eighty-nine per cent of cases reported they had tested HIV-positive, compared with 46% of CGa (p<0.001) and 68% of CGs (p=0.002). More comprehensive details of incident STI diagnoses and clinical presentations have been reported elsewhere.^20^

In the following analyses of sexual behaviour, we exclude two cases and four symptomatic controls (CGs) who, at the time of completing the CASI, reported no sex with another man within the previous 3 months. The remaining 88 cases and 139 controls reported sex exclusively with men during this period.

### Cases compared with asymptomatic controls (CGa)

Cases were significantly more likely to report sex with a greater number of men, with anonymous contacts, meeting men in backrooms, via the internet and at private parties in the previous 3 months (table 2). Cases were significantly more likely to report rectal douching to prepare for sex, with a device used by another and not sterilised in between, sex under the influence of certain recreational drugs, group sex (sex involving more than two men) and water sports (sex play involving urine). Cases were significantly more likely to report unprotected (without condoms) anal intercourse (UAI) with HIV-positive men. Taking into account the HIV status of the respondent, cases were also significantly more likely to report HIV-positive sero-concordant UAI.

**Table 2:** LGV cases compared with asymptomatic controls

Characteristic		Category	Cases (%)	Asymptomatic controls (%)	Univariable			Multivariable		
					OR	95% CI	p Value	AOR	95% CI	p Value
Number			88 (100)	74 (100)						
Role-specific sex acts										
Self	Other									
Mouth	Mouth	Deep kissing	79 (90)	68 (92)	0.8	0.3 to 2.3	0.644			
	Penis	Receptive oral sex (ROS)	86 (98)	72 (97)	1.2	0.2 to 8.7	0.861			
	Penis no condom	Unprotected ROS	84 (98)	70 (95)	2.4	0.4 to 13.5	0.320			
	Anus	Rimming	70 (80)	53 (72)	1.5	0.7 to 3.2	0.242			
Penis	Mouth	Insertive oral sex (IOS)	86 (98)	72 (97)	1.2	0.2 to 8.7	0.861			
	Mouth	Unprotected IOS	86 (98)	67 (91)	4.5	0.9 to 22.3	0.066			
	Anus	Insertive AI (IAI)	78 (89)	61 (84)	1.7	0.7 to 4.3	0.259			
	Anus no condom	Unprotected IAI	74 (85)	33 (46)	6.7	3.2 to 14.2	<0.001			
Anus	Mouth	Being rimmed	81 (92)	62 (85)	2.1	0.8 to 5.6	0.160			
	Finger	Being fingered	68 (77)	52 (71)	1.4	0.7 to 2.8	0.382			
	Sex toy	Toy inserted	36 (41)	12 (16)	3.5	1.7 to 7.5	0.001			
	Hand	Being fisted	19 (22)	1 (1)	19.8	3.0 to 834.8	<0.001*			
	Penis	Receptive AI (RAI)	85 (97)	59 (81)	6.7	1.8 to 24.4	0.004			
	Penis no condom	Unprotected RAI	81 (93)	35 (48)	14.7	5.7 to 37.8	<0.001	10.7	3.5 to 32.8	<0.001
Finger	Anus	Fingering	69 (80)	54 (74)	1.4	0.7 to 3.0	0.348			
Hand	Anus	Fisting another	32 (37)	3 (4)	13.8	3.9 to 73.2	<0.001*	6.7	1.8 to 25.3	0.005
Sex toy	Anus	Inserting toy in other	34 (40)	11 (15)	3.7	1.7 to 8.0	0.001			
General behaviours										
Contacts		More than 10	40 (45)	18 (24)	2.6	1.3 to 5.1	0.006			
Partnership type		Partner or ex	42 (48)	34 (46)	1.1	0.6 to 2.0	0.821			
		Regular	43 (49)	25 (34)	1.9	1.0 to 3.5	0.054			
		Casual	69 (78)	51 (69)	1.6	0.8 to 3.3	0.172			
		Anonymous	50 (57)	22 (30)	3.1	1.6 to 6.0	0.001	2.7	1.2 to 6.3	0.020
Meeting men via		Bar or club	36 (41)	27 (36)	1.2	0.6 to 2.3	0.565			
		Backroom	25 (28)	10 (14)	2.5	1.1 to 5.7	0.024			
		Sauna	35 (40)	23 (31)	1.5	0.8 to 2.8	0.251			
		Cruising ground	15 (17)	14 (19)	0.9	0.4 to 2.0	0.757			
		Internet	66 (75)	36 (49)	3.2	1.6 to 6.2	0.001			
		Private party	28 (32)	12 (16)	2.4	1.1 to 5.2	0.024			
Rectal douching		To prepare for sex	74 (84)	43 (58)	3.8	1.8 to 7.9	<0.001			
〃Equipment		Used by another not sterilised	41 (47)	17 (23)	2.9	1.5 to 5.8	0.002			
Sex under influence		GHB/GBL	50 (57)	15 (21)	5.2	2.6 to 10.6	<0.001	3.1	1.3 to 7.4	0.011
		Viagra	49 (56)	25 (34)	2.5	1.3 to 4.7	0.006			
		Methamphetamine	40 (46)	7 (10)	8.0	3.2 to 22.8	<0.001*			
Group sex		Sex involving > 2 men	65 (49)	32 (24)	3.7	1.9 to 7.2	<0.001			
Water sports		Sex play involving urine	43 (49)	16 (22)	3.5	1.7 to 6.9	<0.001			
Unprotected anal intercourse†		with HIV+	69 (78)	26 (35)	6.7	3.3 to 13.5	<0.001			
		HIV+ with HIV+‡	65 (74)	24 (32)	5.9	3.0 to 11.6	<0.001			
		with HIV status unknown	18 (20)	10 (14)	1.6	0.7 to 3.8	0.247			

This table shows sexual behaviour in previous 3 months.

*Fishers exact.

†Without condoms, either way.

‡Respondent HIV-positive reporting unprotected anal intercourse with HIV-positive.

In terms of role-specific sex acts, 99% of cases and 97% of CGa reported sex of some kind involving their anus in the previous 3 months. Cases were significantly more likely to report receptive anal intercourse (RAI) and unprotected RAI (URAI) (table 2). Of the other activities involving the respondent's anus, cases were significantly more likely to report anoreceptive use of sex toys and being fisted (hand inserted into rectum). Of the sex acts involving the respondent's penis, cases were significantly more likely to report unprotected insertive anal intercourse (UIAI). None of the sex acts involving the respondent's mouth showed significant differences between these groups. Of the sex acts involving the anus of another, in addition to UIAI, insertive use of sex toys and fisting were significantly more common among cases than CGa. Only 7% of cases and 7% of CGa reported no sex involving the anus of another man in the previous 3 months.

Multivariable modelling of general risk behaviours identified anonymous contacts, douching in preparation for sex, and sex under the influence of gamma-hydroxybutyrate or analogues (GHB) as independent risks (data not shown). Multivariable modelling of the role-specific sex acts indicated that URAI, UIA and fisting another were independent risks (data not shown). A combined final model indicated that behavioural risk factors for rLGV were: sex with anonymous contacts (AOR=2.7, 95% CI 1.2 to 6.3), sex under the influence of GHB (AOR=3.1, 95% CI 1.3 to 7.4), URAI (AOR=10.7, 95% CI 3.5 to 32.8) and fisting another (AOR=6.7, 95% CI 1.8 to 25.3) when comparing cases with CGa.

The effect of including HIV status (known HIV-positive OR=9.2, 95% CI 4.1 to 20.5) in this model was explored. The association with HIV was reduced (AOR=6.2, 95% CI 2.9 to 19.5), but remained significant.

### Cases compared with symptomatic controls (CGs)

Similar to comparison with CGa, cases were significantly more likely to report sex with greater numbers of men, rectal douching in preparation for sex, and using unsterilised equipment, sex under the influence of certain recreational drugs, group sex, water sports, UAI with HIV-positive men and HIV-positive sero-concordant UIA, than CGs in the previous 3 months (table 3). Cases were significantly more likely to report sex with casual contacts than CGs.

**Table 3:** LGV cases compared with symptomatic controls

Characteristic		Category	Cases (%)	Symptomatic controls (%)	Univariable			Multivariable		
					OR	95% CI	p Value	AOR	95% CI	p Value
Number			88 (100)	65 (100)						
Role-specific sex acts										
Self	Other									
Mouth	Mouth	Deep kissing	79 (90)	59 (91)	0.9	0.3 to 2.6	0.838			
	Penis	Receptive oral sex (ROS)	86 (98)	62 (95)	2.1	0.3 to 12.8	0.430			
	Penis no condom	Unprotected ROS	84 (98)	60 (95)	2.1	0.3 to 13.0	0.424			
	Anus	Rimming	70 (80)	35 (54)	3.3	1.6 to 6.8	0.001			
Penis	Mouth	Insertive oral sex (IOS)	86 (98)	60 (92)	3.6	0.7 to 19.1	0.135			
	Mouth	Unprotected IOS	86 (98)	58 (89)	5.2	1.0 to 25.9	0.045			
	Anus	Insertive AI (IAI)	78 (89)	36 (55)	7.0	3.0 to 16.3	<0.001			
	Anus no condom	Unprotected IAI	74 (85)	32 (49)	5.9	2.7 to 12.6	<0.001	4.7	2.0 to 10.9	<0.001
Anus	Mouth	Being rimmed	81 (92)	53 (82)	2.6	1.0 to 7.1	0.058			
	Finger	Being fingered	68 (77)	52 (80)	0.9	0.4 to 1.9	0.685			
	Sex toy	Toy inserted	36 (41)	17 (26)	2.0	1.0 to 3.9	0.060			
	Hand	Being fisted	19 (22)	8 (12)	2.0	0.8 to 4.8	0.141			
	Penis	Receptive AI (RAI)	85 (97)	55 (85)	5.2	1.4 to 19.6	0.016			
	Penis no condom	Unprotected RAI	81 (93)	44 (68)	6.4	2.4 to 17.1	<0.001			
Finger	Anus	Fingering	69 (80)	36 (56)	3.2	1.5 to 6.5	0.002			
Hand	Anus	Fisting another	32 (37)	9 (14)	3.6	1.6 to 8.3	0.002	2.0	0.8 to 4.9	0.130
Sex toy	Anus	Inserting toy in other	34 (40)	12 (19)	2.8	1.3 to 6.1	0.007			
General behaviours										
Contacts		More than 10	40 (45)	18 (28)	2.2	1.1 to 4.3	0.026			
Partnership type		Partner or ex	42 (48)	29 (45)	1.1	0.6 to 2.2	0.703			
		Regular	43 (49)	22 (34)	1.9	1.0 to 3.6	0.065			
		Casual	69 (78)	41 (63)	2.1	1.0 to 4.3	0.039			
		Anonymous	50 (57)	31 (48)	1.4	0.8 to 2.7	0.264			
Meeting men via		Bar or club	36 (41)	26 (40)	1.0	0.5 to 2.0	0.910			
		Backroom	25 (28)	10 (15)	2.2	1.0 to 4.9	0.061			
		Sauna	35 (40)	26 (40)	1.0	0.5 to 1.9	0.977			
		Cruising ground	15 (17)	10 (15)	1.1	0.5 to 2.7	0.784			
		Internet	66 (75)	35 (54)	2.6	1.3 to 5.1	0.007			
		Private party	28 (32)	13 (20)	1.9	0.9 to 4.0	0.105			
Rectal douching		To prepare for sex	74 (84)	35 (54)	4.5	2.1 to 9.6	<0.001	2.9	1.3 to 6.6	0.011
〃Equipment		Used by another not sterilised	41 (47)	14 (22)	3.2	1.5 to 6.6	0.002			
Sex under influence		GHB/GBL	50 (57)	21 (32)	2.8	1.4 to 5.5	0.002			
		Viagra	49 (56)	24 (37)	2.2	1.1 to 4.3	0.019			
		Methamphetamine	40 (46)	13 (20)	3.4	1.6 to 7.1	0.001			
Group sex		Sex involving > 2 men	65 (49)	36 (27)	2.3	1.2 to 4.5	0.018			
Water sports		Sex play involving urine	43 (49)	18 (28)	2.5	1.3 to 5.0	0.009			
Unprotected anal intercourse*		with HIV+	69 (78)	29 (45)	4.5	2.2 to 9.1	<0.001			
		HIV+ with HIV+†	65 (74)	26 (40)	4.2	2.1 to 8.4	<0.001			
		with HIV status unknown	18 (20)	11 (17)	1.3	0.6 to 2.9	0.582			

This table shows sexual behaviour in previous 3 months.

*Without condoms, either way.

†Respondent HIV-positive reporting unprotected anal intercourse with HIV-positive.

Regarding role-specific sex acts, cases were significantly more likely to report RAI, URAI, unprotected insertive oral sex, IAI, UIAI and rimming another. Twenty-five per cent of CGs reported no sex of any kind involving the anus of another in the previous 3 months (p=0.004).

In the multivariable model of general behaviours, douching in preparation for sex and UAI with HIV-positive men persisted as independent risk factors (data not shown). In a model combining role-specific sex acts, URAI dropped below statistical significance when included with UIA, which emerged as the only role-specific sex act associated with rLGV comparing cases with CGs.

A final model combining general and role-specific sex acts indicated that rectal douching (AOR=2.9, 95% CI 1.3 to 6.6) and UIAI (AOR=4.7, 95% CI 2.0 to 10.9) were independent behavioural risks comparing cases with CGs (table 3).

The risk of being HIV-positive (OR=3.5, 95% CI 1.5 to 8.1) was examined in this model and fell below statistical significance (AOR=1.9, 95% CI 0.7 to 4.9), with little change in risks of douching (AOR=3.1, 95% CI 1.3 to 6.9) or UIAI (4.4, 95% CI 1.9 to 9.7).

### Potential LGV acquisition events

Potential LGV acquisition events were reported by 41 cases. Thirty-four clarified they were ‘completely’ or ‘fairly’ sure (17, respectively) they had identified the event when LGV had been acquired, and these events are summarised. Being a ‘one-off’ (59%), or finding out later that the other person(s) had LGV (24%), were the main criteria cited for selection.

Ninety-two per cent reported the event as involving casual sexual contacts, 44% of whom were anonymous, and 15% reported multiple partners. All reported RAI and 94% URAI; IAI was reported by 65%, and UIAI 59%. Twenty-one per cent reported having sex toys used on them and 12% being fisted; 15% reported using sex toys and 15% fisting another. Of the 10 who did not report insertive anal sex practices during the event, nine reported that RAI occurred in sex on premises venues or a cruising ground. Common factors cited as contributing to the event were recreational drug use (53%), typically methamphetamine and GHB, followed by being told the other person was HIV-positive (50%)—94% of respondents being HIV-positive themselves, alcohol use (36%) and ‘getting carried away’ (33%).

**Figure 1:**
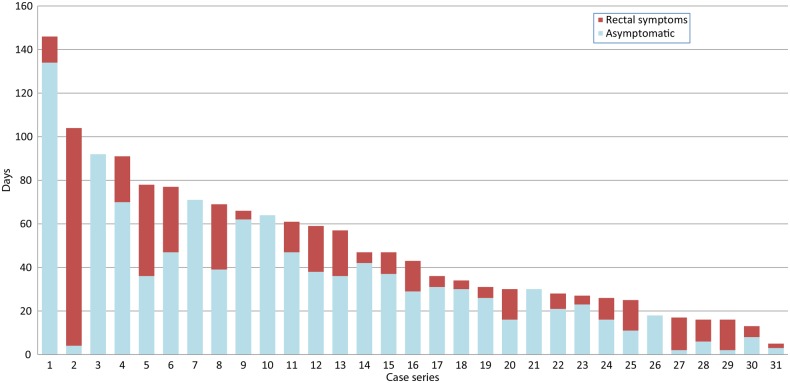
Estimated days from reported event to onset of rectal symptoms and LGV diagnosis. Case series.

Estimating the time elapsed between these episodes (month and year were reported) and presentation at the clinic for LGV diagnosis gave a median delay of 36 days (IQR=25–66). Of these 34 cases, 29 (85%) were recorded as having rectal symptoms on presentation, with a median duration of 17 days (IQR=5–21), although the duration of rectal symptoms was not reported for three. For the remaining 31, data were examined to estimate the incubation period, that is, the time between acquisition and the start of symptoms, showing a median of 30 days (IQR=11–39) for the 26 who had symptoms; the remaining five were asymptomatic at diagnosis (figure 1).

## Discussion

We identified URAI as a key risk factor for rectal LGV infection in MSM, evident in the comparison of rLGV cases with asymptomatic controls. This finding, although not unexpected, supports the hypothesis that rectal infection is due to direct inoculation.^21^ Clearly, this can occur either directly from a man with urethral infection, or indirectly by transfer from another infected rectum on a covered or uncovered penis, sex toy or finger without the insertive partner necessarily having LGV infection. The latter may in part explain the predominance of rectal cases that characterise this outbreak and the latter may account for the independent risks of insertive anal practices observed in this study, particularly fisting, which are capable of facilitating rectal-to-rectal transmission. We also identified a role of sex under the influence of drugs and with anonymous contacts. While we cannot assume that such exposures measured over a three-month period necessarily coincide, they were common features of the accounts of when LGV infection was believed to have been acquired.

That the final multivariable model comparing symptomatic controls identified different risk factors—douching and UIAI—is problematic to interpret and surprising given the overlap of risk factors in the univariable analyses. The divergence may have arisen because a quarter of symptomatic controls did not report any insertive anal practices, compared to 7% of cases and asymptomatic controls. If insertive anal practices provide key opportunities for rectal-to-rectal transmission, then the tendency of symptomatic controls to not practise these behaviours may emphasise the associated risks among cases. This potential bias may be beyond our study's power to control, and may also relate to why douching in preparation for sex, also persisted to the final model comparing symptomatic controls. However, rectal douching is a common practice among men who engage in URAI,^22^ and the risks of either are difficult to separate. A previous study also identified a risk of douching,^16^ perhaps reflecting a similar control group (MSM with non-LGV proctitis).

Our findings are subject to limitations of all case-control studies. Recruiting controls from the same clinics as cases will have minimised selection bias, as will the similar participation rates for the three groups. The use of CASI will have standardised interviewer bias, assisted recall and reporting, but such biases are unavoidable. The shortfall in patient surveys highlights a drawback in home completion; otherwise, the use of CASI appears successful. The accounts of where LGV may have been acquired are speculative but provide insights to the interplay of potential risk factors. The estimate of a median incubation period is tentative, but may be the best estimate to date in the absence of experimental study.

Given that controls were recruited consecutively from the same clinics as cases and were, therefore, broadly matched by HIV status, our study had limited scope to investigate HIV as a risk factor. The risk of being HIV-positive dropped below statistical significance when included in the final CGs model, but remained significant, although diminished, in the CGa model. HIV-positive serosorting appeared to underpin much of the high-risk sexual behaviour described in this study.^23^ Meeting men online, reported by 75% of cases, can provide an efficient means of establishing mutual HIV disclosure,^24^ but can also connect men with specific sexual and recreational drug interests. This can lead to highly assortative mixing patterns capable of connecting dense sexual networks in which LGV circulates.

Our findings support advice that condoms provide protection against LGV, and that particular care is required in group sex situations to prevent pathogens being transferred from the rectum of one man to another.^25^ Rectal-to-rectal transmission is also possible between couples, particularly when insertive and receptive anal sex roles are practised by both parties, evident from the accounts of potential LGV acquisition events in our study. Versatility in unprotected anal sex roles has been recognised as an important component in HIV transmission dynamics of MSM,^26^ and may also be a driver of the current LGV outbreak.

The potential risks to sexual health of the use of recreational drugs within this population and in specific settings/situations, and the challenges these pose to practising safer sex were also evident. Campaigns to raise awareness of LGV and of the symptoms among gay men, particularly HIV-positive men, should be updated and maintained. There should be further discussion about the risks of STIs when HIV-positive men are serosorting. Risk factors for LGV support transmission of other STIs including HIV, hepatitis C, syphilis and gonorrhoea, including antibiotic-resistant strains.^27^ Sexual health clinics should identify men at risk, encourage frequent STI screening, provide adequate treatment and contact tracing, and offer appropriate support to minimise risks associated with sexual behaviour and substance use.

**Key messages**

- Unprotected receptive anal intercourse is a key risk factor for LGV in men who have sex with men.
- Rectal-to-rectal transmission can be facilitated by insertive anal sex practises.
- Men at risk of LGV should be targeted for frequent STI screening and interventions to reduce risks.
